# Development of a UPLC-MS/MS method for the determination of sulfatinib and its no interaction with myricetin in rats

**DOI:** 10.3389/fphar.2024.1498339

**Published:** 2024-12-04

**Authors:** Dongxin Chen, Jie Chen, Hailun Xia, Xiaohai Chen, Jinyu Hu, Guangliang Wu, Xuegu Xu

**Affiliations:** ^1^ The Affiliated Lihuili Hospital, Ningbo University, Ningbo, Zhejiang, China; ^2^ The First Affiliated Hospital of Wenzhou Medical University, Wenzhou, Zhejiang, China; ^3^ The Eye Hospital of Wenzhou Medical University, Wenzhou, Zhejiang, China

**Keywords:** sulfatinib, myricetin, drug-drug interaction, pharmacokinetics, UPLC-MS/MS

## Abstract

**Introduction:**

Sulfatinib is a novel oral tyrosine kinase inhibitor (TKI) with selective inhibition of fibroblast growth factor (FGFR), colony-stimulating factor 1 receptor (CSF-1R) and vascular endothelial growth factor receptor (VEGFR) 1, 2, and 3. It has been approved for the therapy of neuroendocrine tumors arising in the non-pancreatic (December 2020) and pancreatic (June 2021) glands. Until now, there has no research on the determination of sulfatinib in biological medium by ultra performance liquid chromatography tandem mass spectrometry (UPLC-MS/MS) method.

**Methods:**

The current study validated a sensitive and reliable quantitative detection of sulfatinib in plasma using UPLC-MS/MS for the first time, and investigated the interaction with myricetin in rats. Acetonitrile was used to precipitate the plasma protein, and lenvatinib was employed as the internal standard (IS).

**Results:**

The method demonstrated that sulfatinib presented high linearity over the concentration of 11–2,000 ng/mL with the lower limit of quantification (LLOQ) of 1 ng/mL. It was validated methodologically that the precision, matrix effect, stability, accuracy and extraction recovery were all within the allowable values. Moreover, male Sprague-Dawley (SD) rats were assigned randomly to assess the interaction between sulfatinib (30 mg/kg) and myricetin (50 mg/kg). Nevertheless, no significant differences of the main pharmacokinetic parameters were revealed. This may be due to insufficient doses of myricetin, or failure of myricetin to act in a timely manner *in vivo*.

**Discussion:**

The findings contributed to a better understanding of the metabolism and drug-drug interaction of sulfatinib, but the presence or absence of interactions needs to be confirmed by further studies.

## 1 Introduction

In many types of tumors, such as uroepithelial carcinoma, cholangiocarcinoma and carcinoma, fibroblast growth factor receptor (FGFR) activating mutations, gene amplification, gene rearrangements or fusions have been observed (Dieci et al., 2013; Qu et al., 2022). Differentiation, proliferation and survival of mononuclear phagocytes are regulated by macrophage colony-stimulating factor receptor (CSF1R) signaling, which is active in the microenvironment of many tumors thus contributing to drug resistance and immune evasion (Chitu and Stanley, 2006; Morrison, 2016; Mantovani et al., 2017). In addition, the upregulation of vascular endothelial growth factor receptors (VEGFR) in response to changes in the FGFR gene promotes tumor angiogenesis (Giavazzi et al., 2003; Zhang et al., 2020).

Sulfatinib is selective in inhibiting FGFR, CSF-1R and VEGFRs (VEGF1, 2, 3), making it a novel oral tyrosine kinase inhibitor (TKI) (Lin et al., 2024). It has been approved for the therapy of neuroendocrine tumors arising in the non-pancreatic (December 2020) and pancreatic (June 2021) glands. After oral administration of sulfatinib, the serum level of the original drug is about 40.3%. N-demethylation, indolyl cyclomethylation, glucuronidation and sulfation are important metabolic modes *in vivo*. Enzymatic phenotyping studies *in vitro* have demonstrated that cytochrome P450 3A4 (CYP3A4) is the major enzyme mediating the metabolism of sulfatinib (Li et al., 2020b). The excretion route is mainly fecal (88%) (Lu et al., 2024; Xu et al., 2017; Li et al., 2020a).

Side effects of sulfatinib treatment have been reported as hypertension, proteinuria, bleeding, liver injury, hyperuricemia, hypertriglyceridemia, and diarrhoea (Manjulika and Das, 2019; Cao et al., 2020; Xu et al., 2020a). High blood pressure is usually grade 1 to 2 and usually occurs within 2 weeks.

Myricetin is a flavonoid compound found in abundance in teas, red wines, berries, vegetables and herbs with a multitude of pharmacological effects such as antioxidant, cardiovascular protection, anti-inflammatory, hepatoprotective activity and antitumor properties (Ong and Khoo, 1997; Gupta et al., 2020a; Gupta et al., 2020b). Studies have demonstrated that myricetin is also protective against kidney injury (e.g., cisplatin-induced acute kidney injury and diabetic kidney injury) (Qi et al., 2024; Yang et al., 2019). Clinically, the combination of myricetin with sulfatinib may be used to ameliorate sulfatinib-induced renal injury as well as hepatic injury. Nevertheless, the studies in rats suggested that myricetin inhibited CYP3A4 and CYP2C9, which significantly reduced the AUC and C_max_ of finasteride (Li et al., 2018). For this reason, it is important to exercise caution when combining myricetin with CYP3A4 or CYP2C9 substrates.

As we known, sulfatinib is mainly metabolized by CYP3A4, and myricetin is an inhibitor of CYP3A4. Drug-drug interactions (DDI) may occur when sulfatinib and myricetin are used in combination, which may cause an increase in the incidence of adverse events with sulfatinib or even threaten the patient’s life. However, there has been no documented DDI between sulfatinib and myricetin. Furthermore, to date, there are no published studies investigating the DDI of sulfatinib.

Currently, there was only one publication that briefly mentioned the use of UPLC to detect the concentration of sulfatinib, but the methodological validation and sample processing were not mentioned (Li et al., 2020b). Therefore, it can be considered there is no bioanalytical assay for the quantification of sulfatinib in biological media to characterize the pharmacokinetic profile, DDI and therapeutic drug monitoring studies. Hence, the aim of the research was to develop and validate a ultra performance liquid chromatography tandem mass spectrometry (UPLC-MS/MS) approach of reliability and sensitivity for the quantitative detection of sulfatinib and to investigate the interaction between sulfatinib and myricetin *in vivo*.

## 2 Materials and methods

### 2.1 Reagents and chemicals

**FIGURE 1:**
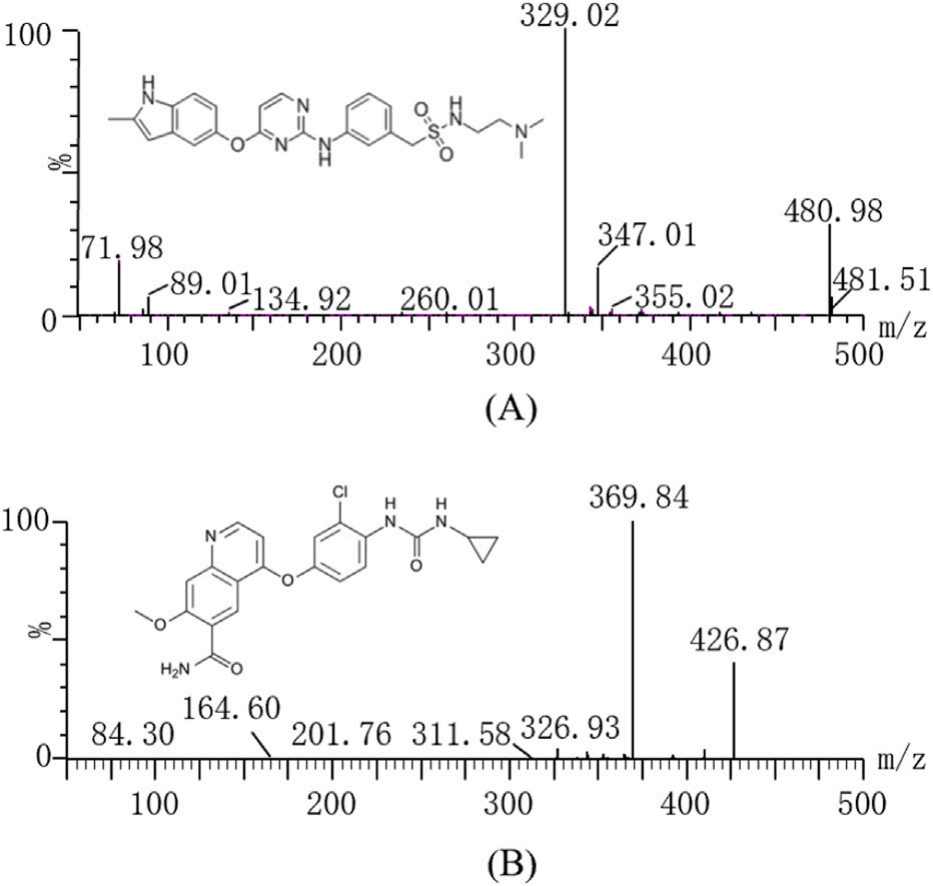
The chemical structures and product mass spectra of sulfatinib **(A)**, and lenvatinib **(B)**.

Sulfatinib (Figure 1A, over 99% purity) and lenvatinib (Internal standard, IS, over purity 99%, Figure 1B) were obtained from Shanghai Canspec Scientific Instruments Co., Ltd. (Shanghai, China). Myricetin (purity over 99%) was also bought from Shanghai Canspec Scientific Instruments Co., Ltd. (Shanghai, China). Ultrapure water for the formulation of mobile phase and solution was prepared by an ultrapure water unit from Millipore Corporation (Bedford, United States). Methanol and acetonitrile for chromatography were bought from Merck (Darmstadt, Germany). All other solvents and chemicals employed in the investigations were of analytical grade.

### 2.2 Experimental apparatus and conditions

In the present assay, a Waters Xevo TQS triple quadrupole tandem mass spectrometer (Milford, MA, United States), connected to a Waters Acquity UPLC I class system (Milford, MA, United States) was applied in the method. The parameters of mass spectrometry were determined as follows before analysis: capillary voltage 2.0 kV, desolvation gas 1000 L/h, collision gas 0.15 mL/min, desolvation temperature 600°C and conical gas 150 L/h. Seperations of sulfatinib and IS were done on an Acquity UPLC BEH C18 (2.1 mm × 50 mm, 1.7 µm) set at 40°C. The autosampler used to analyze all samples was held at 10°C. The measurements were conducted using a multiple reaction monitoring (MRM) scanning mode with an electrospray ionization (ESI) source in positive ion mode. At a flow rate of 0.40 mL/min, the mobile phase was consisted of acetonitrile (A) and 0.1% formic acid aqueous solution (B), with a linear gradient scheme as follows: 0–0.5 min, A/B = 10/90%; 0.5–1.0 min, A/B = 90/10%; 1.0–1.4 min, A/B = 90/10%; 1.4–1.5 min, A/B = 10/90%; 1.5–2.0 min, A/B = 10/90%. The parameters optimized for mass spectrometry of sulfatinib and IS included cone voltages of 20 V and 30 V, and collision energies of 25 eV and 28 eV, respectively. The parent and daughter ions of sulfatinib and IS were *m/z* 480.98 → 329.02 and *m/z* 426.87 → 369.84, respectively (as indicated in Table 1).

**TABLE 1:** Specific mass spectrometric parameters and retention times (RTs) for sulfatinib and IS, including cone voltage (CV), and collision energy (CE).

Analytes	Precursor ion	Product ion	CV (V)	CE (eV)	RT (min)
Sulfatinib	480.98	329.02	20	25	1.21
IS	426.87	369.84	30	28	1.16

### 2.3 Calibration solution and quality control samples (QCs) preparation

Stock solution was formulated by dissolving sulfatinib or IS in methanol up to a concentration of 1.00 mg/mL. After that, different concentrations of working solutions were made by gradual dilution with methanol. Calibration samples of sulfatinib were obtained by pipetting 10 µL of the working solution into 90 µL of blank plasma to obtain samples of sulfatinib (1, 5, 10, 50, 100, 500, 1,000 and 2,000 ng/mL). The four QCs of sulfatinib (1, 2, 800, and 1,600 ng/mL) were formulated in the same way. The concentration of IS (lenvatinib) working solution was 200 ng/mL. Both stock and working solutions were formulated and aliquoted in 1.5 mL polypropylene tubes, and stored at −80°C in advance.

### 2.4 Sample processing

The protein was precipitated by mixing 300 µL of acetonitrile and 10 µL of IS working solution into 100 µL of plasma. After a full vortex for 2.0 min, the mixture was centrifuged at 4°C for 10 min (13,000 rpm). Afterwards, the supernatant of 100 µL was moved into a vial, with a 2 µL injected and analyzed by UPLC-MS/MS.

### 2.5 Method validation

The validation assay was performed based on FDA criteria, including lower limit of quantification (LLOQ), selectivity, precision, linearity, accuracy, matrix effect, stability and extraction recovery (Tang et al., 2022).

#### 2.5.1 Selectivity

Specificity describes the ability of an analytical approach to correctly determine the target compound when other components may be present. By examining three chromatograms of blank plasma, standard solution, and post-oral rat plasma samples from six different rats, the selectivity of the quantification was determined.

#### 2.5.2 Sensitivity and linearity

There were 8 non-zero samples to prepare the calibration curve. The peak area ratio of analyte/IS was plotted against the concentration of the analyte by weighted (*1/x* ^*2*^) least squares regression analysis. A typical regression equation is y = kx + b, where y is peak area ratio of the analyte/IS, x is the concentration of analyte in plasma sample, and the correlation coefficient (*r* ^*2*^) needs to be higher than 0.99. The least detectable amount of sample is the lower limit of quantification (LLOQ), which needs to meet certain precision and accuracy requirements (accuracy was between 80% and 120%, and precision was ≤20%).

#### 2.5.3 Accuracy and precision

Samples of rat plasma with four different concentrations (1, 2, 800 and 1,600 ng/mL) of QCs were assayed for 1 day and three consecutive days. The relative standard deviation (RSD%) and relative error (RE%) were determined whether the values were within the required ranges. The RE% was in the range of 85%–115% and the RSD% was less than 15% for the three QCs except LLOQ.

#### 2.5.4 Matrix effect and extraction recovery

Assessment of extraction recovery and matrix effect was performed using blank plasma from different rats and three levels of QCs (2, 800 and 1,600 ng/mL). For the evaluation of the recovery, the peak areas of the pre-spiked QCs were compared with the peak areas of the spiked samples from blank plasma after extraction. The matrix effect was obtained by comparing the peak area ratio of blank plasma treated with the addition of work solutions and neat solutions at the same concentration.

#### 2.5.5 Stability

The stability was investigated by repeating the assay five times under different storage conditions for three rat plasma QCs (2, 800 and 1,600 ng/mL). The conditions included stability in the analyzer (4 h, 10°C), long-term storage (80°C, 3 weeks), short-term storage (3 h, room temperature) and freeze-thaw cycles (three complete times).

### 2.6 DDI study

Ten male Sprague-Dawley (SD) rats for this experiment, weighting 200 ± 10 g, were purchased from the Experimental Animal Center of The First Affiliated Hospital of Wenzhou Medical University (Zhejiang, China). Prior to the start of the investigation, all rats were domesticated under laboratory conditions for 1 week to minimize their suffering. The animals were kept in an appropriate environment (temperature, humidity, light conditions, freely available food and water). The experimental process of the animals was strictly adhered to the regulations for the care and use of laboratory animals as reviewed and approved by the Ethics Committee of The First Affiliated Hospital of Wenzhou Medical University (Wenzhou, China). The preparation of sulfatinib for gavage was carried out using 0.5% carboxymethylcellulose sodium (CMC-Na) solution. Half an hour after the administration of either myricetin or the same dose of 0.5% CMC-Na solution, 10 rats were simultaneously administered orally with sulfatinib (30 mg/kg). At 0.25, 0.5, 0.75, 1, 1.5, 2, 3, 4, 6, 8, 12, 24, 48 h, approximately 0.3 mL of blood was obtained from the tail vein of SD rats and transferred into heparinized polyethylene tubes. A 100 µL of plasma was obtained by centrifuging the blood at 13,000 rpm for 8 min and stored at −80°C for sample treatment.

### 2.7 Statistical analysis

Origin 9.0 (Originlab Inc., Northampton, MA, United States) was used in this experiment to plot the mean concentration of sulfatinib versus time in plasma. DAS software (Drug and Statistics, Version 3.0, China) was applied to fit the non-compartmental model of sulfatinib, and the main pharmacokinetic parameters of sulfatinib were obtained. Pharmacokinetics were compared for each group using SPSS software (version 24.0, Chicago, United States, SPSS Inc.) using the independent samples *t*-test. At *p* < 0.05, the difference was significant.

## 3 Results

### 3.1 Method development and optimization

It was optimized for the mode of elution, mobile phase, column temperature and column to achieve better efficient separation of sulfatinib and IS in rat plasma. Aqueous phase (water, 0.1% formic acid, 0.1% acetic acid, and 1 mM ammonium acetate buffer) and organic phase (such as acetonitrile and methanol) with different proportions mixed were assessed. Given that adding formic acid into mobile phase in a linear gradient scheme might improve the ionization efficiency and sensitivity of sulfatinib and IS, the final suitable mobile stage was chosen as the combination of acetonitrile and water (containing 0.1% formic acid). In addition, different LC columns at various temperatures were investigated and the comparisons of chromatographic separation were carried out. Finally, a BEH C18 column at 40°C was chosen due to high separation efficiency, lower background noise and good peak symmetry. In this study, the extraction recovery, precision, accuracy, stability, and matrix effect of the method under above conditions were assessed with the results in the acceptable ranges.

### 3.2 Method validation

#### 3.2.1 Selectivity

**FIGURE 2:**
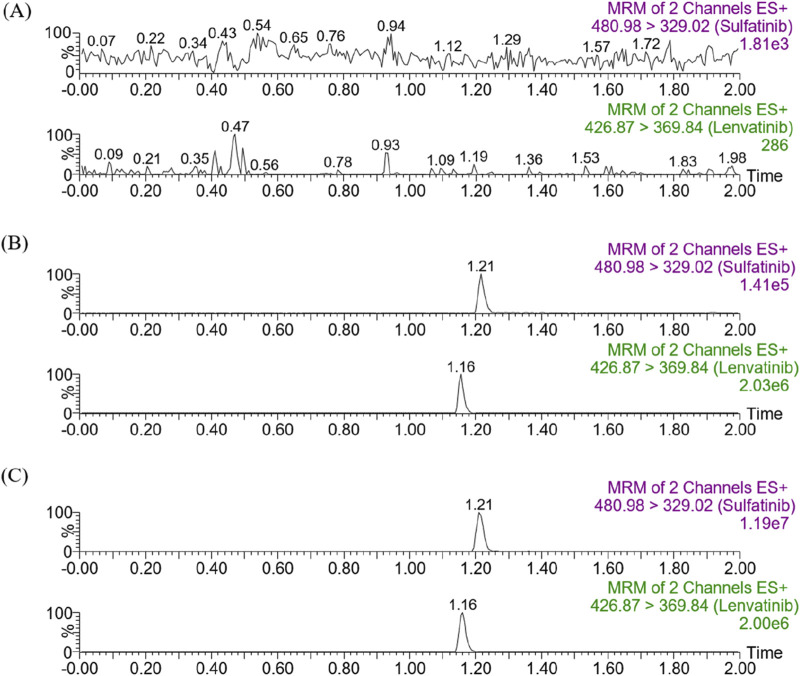
Representative chromatograms of sulfatinib and IS in SD rat plasma: **(A)** blank plasma; **(B)** blank plasma spiked with analytes at LLOQ; **(C)** sample obtained from a rat at 1.0 h after oral administration of 30 mg/kg sulfatinib.

Figure 2 showed the chromatograms of blank plasma samples from rats (A), plasma samples spiked with analytes at LLOQ and IS (B), and plasma sample from rat 1.0 h after receiving an oral dose of 30 mg/kg of sulfatinib (C). Retention times were observed to be 1.21 min and 1.16 min for sulfatinib and IS, respectively. There were no interferences by endogenous substances or other impurities in the method. It was demonstrated that the approach for the quantification of sulfatinib in plasma had good selectivity and specificity.

#### 3.2.2 Linearity and sensitivity

There was a high degree of linearity in the calibration curve over the range of 1–2,000 ng/mL with the equation of y = 0.0496636 × x + 0.808537 (*r* ^*2*^ = 0.999). The LLOQ for sulfatinib was 1 ng/mL, which showed an accuracy of less than ±20% and a precision of less than 20% (as revealed in Table 2) (Guidance for industry, 2018).

**TABLE 2:** Intra- and Inter-day accuracies and precisions of sulfatinib in rat plasma (n = 5).

Compound	Concentration (ng/mL)	Intra-day		Inter-day	
		RSD%	RE%	RSD%	RE%
	1	15.5	−10.7	12.9	−3.6
Sulfatinib	2	7.3	−0.6	7.0	−0.4
	800	6.0	0.9	6.9	−1.7
	1,600	2.8	−10.3	5.7	−5.0

#### 3.2.3 Inter and intra-day precision and accuracy

Inter- and intra-day accuracy and precision of sulfatinib were quantified at four levels (1, 2, 800, and 1,600 ng/mL) on three consecutive days, as summarized in Table 2. The inter and intra-day accuracies of sulfatinib were between −10.7% and 0.9%, with precision of less than 15.5%. The experimental data demonstrated that the analytical method had been characterized by excellent reproducibility and accuracy.

#### 3.2.4 Matrix effect and recovery

The extraction recovery and matrix effect of sulfatinib were summarized in Table 3. The recoveries of sulfatinib in rat plasma at three QCs concentrations were from 97.1% to 114.1%, and the matrix effect was in the range of 108.7%–113.3%. Both of them were in accordance with the standard range (85%–115%). These findings demonstrated that the recoveries of the approach were excellent and the influence of matrix effect could be negligible in routine assays.

**TABLE 3:** Recovery and matrix effect of sulfatinib in rat plasma (n = 5).

Analytes	Concentration (ng/mL)	Recovery (%)		Matrix effect (%)	
		Mean ± SD	RSD (%)	Mean ± SD	RSD (%)
	2	114.11 ± 11.1	9.7	113.3 ± 9.9	8.7
Sulfatinib	800	97.1 ± 5.4	5.6	113.2 ± 5.6	5.0
	1,600	107.2 ± 9.4	8.8	108.7 ± 5.7	5.2

#### 3.2.5 Stability

The stability experiments were evaluated from diverse preserved and processing environments. The conditions of this study included stability in the analyzer (10°C for 4 h), −80°C for 3 weeks, room temperature for 3 h and freeze-thaw cycles (three complete times). The results indicated that different stability experiments were satisfied.

### 3.3 DDI between sulfatinib and myricetin in rats

**FIGURE 3:**
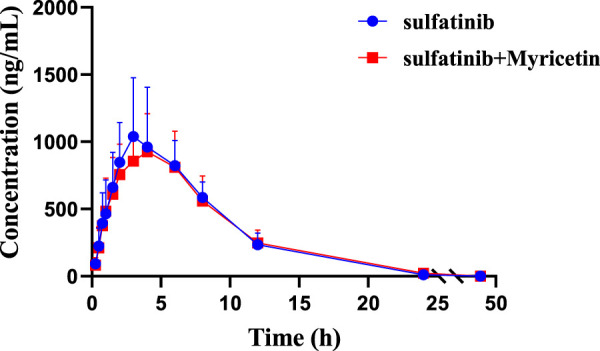
Mean plasma concentration-time curves of sulfatinib in two rat groups (Group A: 30 mg/kg sulfatinib dosed orally alone; Group B: 30 mg/kg sulfatinib and 50 mg/kg myricetin dosed orally). (n = 5, Mean ± SD).

The concentration of sulfatinib in rat plasma was successfully examined by firstly established a UPLC-MS/MS analytical approach, and pharmacokinetic data were obtained from different groups. Figure 3 illustrated the mean plasma concentration versus time curves after a single gavage of 30 mg/kg sulfatinib alone and concomitant gavage of 50 mg/kg myricetin in rats. Table 4 summarized the basic pharmacokinetic parameters calculated under the non-compartmental model.

**TABLE 4:** The main pharmacokinetic parameters of sulfatinib in two SD rat groups (Group A: 30 mg/kg sulfatinib dosed orally alone; Group B: 30 mg/kg sulfatinib and 50 mg/kg myricetin dosed orally) (n = 5, Mean ± SD).

Parameters	Sulfatinib	Sulfatinib + myricetin
AUC_0→t_ (ng/mL·h)	9,253.9 ± 2024.9	9,168.6 ± 2,531.4
AUC_0→∞_ (ng/mL·h)	9,256.6 ± 2027.1	9,171.6 ± 2,532.4
t_1/2_ (h)	3.6 ± 0.6	3.6 ± 0.9
T_max_ (h)	3.4 ± 1.7	4.4 ± 0.9
CL_z/F_ (L/h/kg)	3.3 ± 0.6	3.5 ± 1.1
C_max_ (ng/mL)	1,135.0 ± 327.4	957.0 ± 265.3

AUC: area under the plasma concentration-time curve; t_1/2_: elimination half time; T_max_: peak time; CL_z/F_: plasma clearance; C_max_: maximum plasma concentration.

After a single oral intake of 300 mg of sulfatinib in healthy subjects, a C_max_ of 205 ng/mL and an AUC of 2,667 hmg/mL were achieved in approximately 2 h, with a t_1/2_ of 17.1 h (Li et al., 2020a). In the current investigation, after a single oral dose of 30 mg/kg sulfatinib, the AUC and t_1/2_ were 8,971 hmg/mL and 3.64 h, respectively. After approximately 3.8 h, C_max_ reached 1,039.81 ng/mL. There were differences in the pharmacokinetics of sulfatinib for healthy subjects and rats. According to the data of the present study, myricetin had no significant effect on ${AUC}_{0\to \infty }$ and C_max_ of sulfatinib (P > 0.05), and the mean plasma concentration-time curves of Group A and B basically overlapped.

## 4 Discussions

Although there was a paper that mentioned the UPLC detection for sulfatinib, no methodological validation was performed and the sample preparation was not mentioned (Li et al., 2020b). In the present research, the mobile phase composition, elution pattern and column temperature were developed to achieve efficient separation of sulfatinib and endogenous impurities within only 2 min. Moreover, perfect peak shapes and retention values were obtained. And in accordance with previous experiences, the use of acetonitrile for the precipitation of plasma proteins also yielded high recoveries.

In a randomized double-blind trial, monotherapy with sulfatinib significantly extended progression-free survival in patients with advanced neuroendocrine tumors (NETs) at a daily dose of 300 mg (Xu et al., 2020b). Myricetin, a component of traditional Chinese medicine known for its kidney repair properties, holds great therapeutic potential when used in combination with sulfatinib. Regarding the metabolic processes *in vivo*, there are five pathways for myricetin, including reduction, dehydroxylation, methylation, glucuronidation, and sulfation (Gupta et al., 2020a), and it is an effective inhibitor of CYP3A4 (Xu et al., 2023). Preclinical testing of sulfatinib has shown that the metabolism of sulfatinib *in vivo* processes include N-demethylation, indole cyclomethylation, glucuronidation, mono-oxidation, or sulfation. In metabolism, sulfatinib is mainly metabolized by CYP3A4, and myricetin is an inhibitor of CYP3A4.

According to previous studies, myricetin can exert different types of inhibition on tofacitinib *in vitro* through CYP2C19 and CYP3A4, demonstrating moderate inhibitory effects (Ye et al., 2024). In other *in vivo* studies conducted in rats, myricetin significantly altered the pharmacokinetic parameters of ticagrelor, including AUC and C_max_, and exerted a notable impact on the metabolism of ticagrelor both *in vivo* and *in vitro* (Wang et al., 2024). Furthermore, the antitumor effects of traditional Chinese medicine are well recognized, and their lower toxicity compared to chemotherapy drugs greatly increases the potential for interactions with pharmaceuticals (Zhao et al., 2023). Given this analysis, there is a potential for DDI between myricetin and sulfatinib, highlighting the importance of investigating the effect of myricetin on the metabolism of sulfatinib in rats.

However, from the findings of this study, the metabolic processes *in vivo* of the two medications did not significantly interact with each other, and there are few literature to further the process *in vivo.* The reasons for the negative results may be that the dose of myricetin was insufficient to achieve the inhibitory effect, and the differences in drug interactions *in vitro* and *in vivo* were not considered. Moreover, myricetin was administered half an hour in advance, which may not be able to work in time. In conclusion, this study demonstrates that the combination of myricetin and sulfatinib can be safely co-administered without the need for dose adjustments. Given the current limited number of animals in the DDI study, validation of the results is still needed in further studies.

## 5 Conclusion

In this experiment, an ultra-sensitive and accurate UPLC-MS/MS method was established firstly for the determination of sulfatinib in rat plasma. The developed method was carefully validated under FDA guidelines. Although the results did not indicate the effects of myricetin on the metabolism of sulfatinib, it was necessary to confirm the findings with further experiments.

## Data Availability

The original contributions presented in the study are included in the article/supplementary material, further inquiries can be directed to the corresponding authors.
